# Novel Molecular Insights into the Anti-Inflammatory and Antifibrotic Effects of Dexamethasone on Human Ligamentum Flavum-Derived Cells

**DOI:** 10.3390/ijms27073047

**Published:** 2026-03-27

**Authors:** Alfonso Cordero-Barreal, Djedjiga Ait Eldjoudi, Mariam Farrag, Laura González-Blanco, Maximo Alberto Diez-Ulloa, Miguel Ángel González-Gay, Raquel Largo, Francisca Lago, Yousof Farrag, Jesus Pino, Oreste Gualillo

**Affiliations:** 1 IDIS (Instituto de Investigación Sanitaria de Santiago), C027 NEIRID Lab (Neuroendocrine Interactions in Rheumatology and Inflammatory Diseases), Research Laboratory 9, Santiago University Clinical Hospital, Trav Choupana s/n, 15706 Santiago de Compostela, Spain; sitoalcorba@gmail.com (A.C.-B.); djidji.aiteldjoudi@gmail.com (D.A.E.); mariam.r.farrag@gmail.com (M.F.); laurablanco94@hotmail.com (L.G.-B.); jesus.pino@usc.es (J.P.); 2 Tissue Engineering for Orthopaedics and Mechanobiology, Bone & Joint Program, Department for BioMedical Research (DBMR), University of Bern, 3008 Bern, Switzerland; 3 Division of Orthopaedics and Traumatological Surgery, Santiago University Clinical Hospital, 15706 Santiago de Compostela, Spain; maximoalberto.diez@usc.es; 4 Division of Rheumatology, Fundación Jiménez-Diaz Instituto de Investigación Sanitaria, 28006 Madrid, Spain; miguelaggay@hotmail.com (M.Á.G.-G.); rlargo@fjd.es (R.L.); 5 Department of Medicine and Psychiatry, University of Cantabria, 39006 Santander, Spain; 6 SERGAS (Servizo Galego de Saude) and IDIS (Instituto de Investigación Sanitaria de Santiago), Molecular and Cellular Cardiology Lab, Research Laboratory 7, Santiago University Clinical Hospital, 15706 Santiago de Compostela, Spain; francisca.lago.paz@sergas.es; 7 Centro de Investigación Biomédica en Red de Enfermedades Cardiovasculares (CIBERCV), Instituto de Salud Carlos III, 28220 Madrid, Spain; 8 Department of Surgery and Medical Surgery Specialties, University of Santiago de Compostela, 15705 Santiago de Compostela, Spain; 9 SERGAS (Servizo Galego de Saude), Área Sanitaria de Santiago de Compostela e Barbanza, Hospital Clínico Universitario de Santiago de Compostela, 15706 Santiago de Compostela, Spain

**Keywords:** lumbar spinal stenosis, ligamentum flavum hypertrophy, inflammation, fibrosis, dexamethasone

## Abstract

Lumbar spinal stenosis (LSS) is caused by multiple degenerative changes including the hypertrophy of the ligamentum flavum (LFH). Inflammation and fibrosis contribute to LFH and glucocorticoid drugs (GCDs) are generally used to manage LSS symptoms. However, a thorough understanding of the molecular mechanisms exerted by GCD in ligamentum flavum (LF) cells remains incomplete. Primary human LF cells were isolated from surgical specimens and stimulated with pro-inflammatory agents (IL-1α, IL-1β, LPS) or the profibrotic cytokine TGFβ1, in the presence or absence of dexamethasone. Gene and protein expression levels of inflammatory, fibrotic, and ossification-related markers were analysed using RT-qPCR and Western blotting. Dexamethasone significantly suppressed the expression of key pro-inflammatory, fibrotic, and ossification markers (*IL-6*, *COX2*, *COL3A1*, *MMPs*, *TNFRSF11b*) in both acute and prolonged models of LF inflammation. However, under TGFβ1 stimulation, dexamethasone attenuated inflammatory gene expression but failed to reduce the expression of major fibrosis-associated genes, such as *COL3A1*, *bFGF*, and *POSTN*. Dexamethasone effectively suppresses inflammation-mediated fibrosis in LF-derived cells, indicating its potential to both prevent and reverse LFH progression in the context of LSS. However, its limited efficacy against TGFβ1-driven fibrotic pathways highlights the need for combination therapies targeting both inflammation and fibrosis for more comprehensive management of LFH. These findings support further exploration of corticosteroids as therapeutic agents for hypertrophic ligament disorders.

## 1. Introduction

Lumbar spinal stenosis (LSS) is a degenerative condition that is particularly common among the elderly and characterised by lower back and leg pain, numbness, and intermittent claudication, all of which substantially impair the quality of life of those affected. It is a leading cause of morbidity and work-related disability in older adults [1]. A key structural factor contributing to the narrowing of the spinal canal in LSS is hypertrophy of the ligamentum flavum (LFH), which frequently occurs alongside intervertebral disc degeneration and facet joint arthrosis [2].

The ligamentum flavum (LF) is located in the posterior and lateral walls of the spinal canal and is mainly composed of elastic fibres (80% of extracellular matrix) [3]. During LFH, a series of chronic changes occur within the tissue, which results in increased thickness and collagen levels, decreased elastic fibre levels, and the appearance of local calcifications. These alterations result in canal stenosis and severe myelopathies, as the enlarged LF compresses the spinal cord and nerves [4,5,6].

Recent studies have suggested that LFH results from chronic and recurrent tissue inflammation, which ultimately leads to fibrosis [1,7,8]. This inflammatory process can be triggered by several factors such as increased mechanical and oxidative stress [9,10], or the extravasation of immune system cells into the tissue [11]. During inflammation and fibrosis of the LF, there is an increase in the synthesis of collagen, metalloproteases, and TGFβ1, leading to tissue hypertrophy [12].

Currently, the treatment of canal stenosis depends on the severity of the disease, with surgical treatment (laminectomy) eligible for people with severe pain or morbidity. In less advanced stages, non-invasive therapies are usually used, including physiotherapy, lumbar traction, anti-inflammatory drugs, or epidural corticosteroid injections [13,14].

Corticosteroids are widely used as anti-inflammatory and immunosuppressive drugs to treat various conditions, including rheumatoid arthritis, lupus, asthma, and organ transplant rejection [15]. Interestingly, epidural steroid injections were demonstrated to reduce pain scores and Oswestry Disability Index (ODI) scores in 60% of LSS patients [16]. However, the molecular mechanisms underlying the action of glucocorticoids in LFH are still largely unknown.

Therefore, the aim of this study was to evaluate the molecular effects of dexamethasone on both inflammatory and fibrotic pathways in human LF-derived cells.

## 2. Results

### 2.1. Analysis of Anti-Inflammatory Effect of Dexamethasone in Ligamentum Flavum Cells

**Figure 1:**
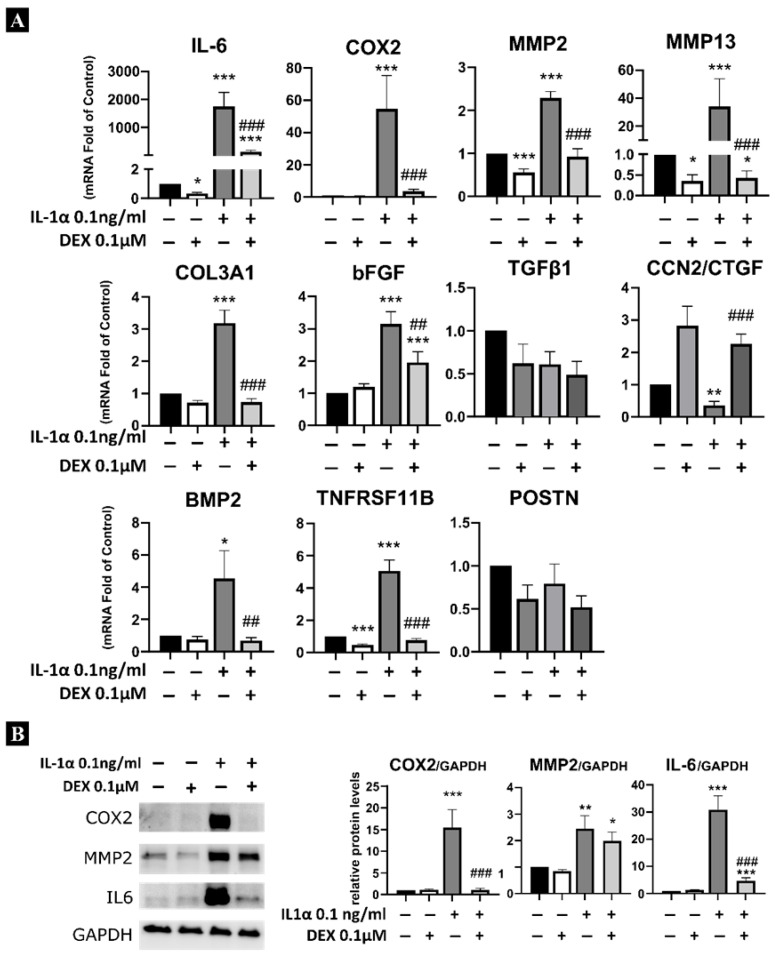
Effect of dexamethasone on the stimulated ligamentum flavum-derived cells (LFCs). mRNA (**A**), measured by rt-qPCR, and protein (**B**), measured by Western blot, 24 h after treatment with IL-1α (0.1 ng/mL) and/or dexamethasone (0.1 µM). Statistical significance is represented as follows: * *p* < 0.05, ** *p* < 0.01, *** *p* < 0.001 compared with control expression levels; ## *p* < 0.01, ### *p* < 0.001 compared with IL-1α treatment.

As shown in Figure 1, stimulation of LF derived cells (LFC) with 0.1 ng/mL IL-1α for 24 h upregulated the mRNA expression of inflammation-associated genes such as *IL-6*, *COX2* and *ELF3*, extracellular matrix (ECM) remodelling genes such as *MMP2* and *MMP13*, hypertrophy-related genes such as *COL3A1* and *bFGF*, and ossification-associated genes such as *BMP2* and *TNFRSF11b*. Conversely, a significant reduction in the expression of *CCN2/CTGF* was observed. No significant differences were observed in *TGFβ1* and *POSTN* gene expressions following IL-1α treatment.

LF cells treated with a combination of IL-1α (0.1 ng/mL) and 0.1 µM of dexamethasone showed significant modulation in the mRNA expression of all the aforementioned upregulated genes. Interestingly, *CCN2* exhibited a distinct response: while IL-1α alone reduced its expression, dexamethasone alone induced *CCN2*. Moreover, the combination of dexamethasone and IL-1α also resulted in *CCN2* upregulation, effectively counteracting the IL-1α-induced reduction. Western blot analysis further corroborated these findings, showing a significant downregulation in the protein expression of IL-6 and COX2 (Figure 1B). While the protein expression of MMP2 also decreased, this reduction was not statistically significant. Notably, comparable outcomes were observed when assessing dexamethasone’s capacity to suppress prolonged inflammation during a 7-day co-treatment with IL-1α (Figure S2).

**Figure 2:**
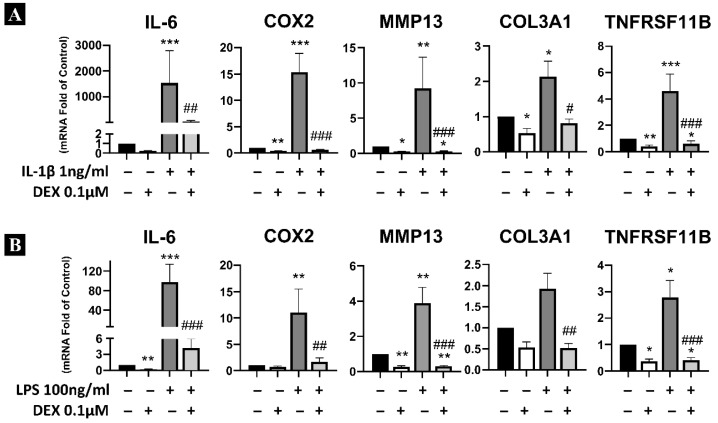
Study of the effect of dexamethasone on inflammatory response with different pro-inflammatory agents. (**A**) Analysis of the effect of dexamethasone on gene expression (mRNA) in the inflammatory response induced by IL-1β (1 ng/mL). (**B**) Analysis of the effect of dexamethasone on gene expression (mRNA) in the inflammatory response induced by LPS (100 ng/mL). Statistical significance is represented as follows: * *p* < 0.05, ** *p* < 0.01, *** *p* < 0.001 compared with control expression levels; # *p* < 0.05, ## *p* < 0.01, ### *p* < 0.001 compared with IL-1β or LPS treatment.

As shown in Figure 2, dexamethasone produced similar effects when combined with other pro-inflammatory agents such as IL-1β (Figure 2A) and LPS (Figure 2B). It significantly downregulated the mRNA expression of pro-inflammatory genes (*IL-6*, *COX2*), the metalloprotease *MMP13*, and markers associated with hypertrophy and ossification (*COL3A1*, *TNFRSF11b*) in inflamed LFCs.

### 2.2. Analysis of the Anti-Inflammatory Effect of Dexamethasone in Long Term-Inflamed Ligamentum Flavum Cells

**Figure 3:**
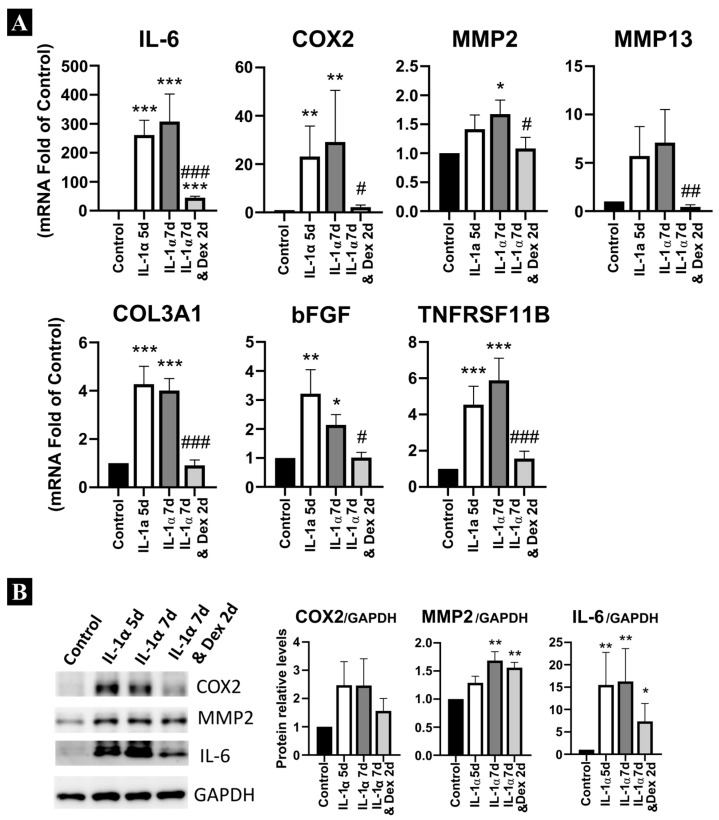
Analysis of the effect of dexamethasone on prolonged inflammation in ligamentum flavum-derived cells. Cells were pretreated with IL-1α for 5 days and then treated with IL-1α in the presence or absence of dexamethasone for two additional days. Expression of mRNA (**A**) and protein (**B**) was analysed. Statistical significance is represented as follows: * *p* < 0.05, ** *p* < 0.01, *** *p* < 0.001 compared with control expression levels; # *p* < 0.05, ## *p* < 0.01, ### *p* < 0.001 compared with IL-1α treatment.

To mimic a state of chronic inflammation, LF cells were exposed to 0.1 ng/mL IL-1α for five days. Subsequently, dexamethasone was introduced at a concentration of 0.1 µM for an additional two days, while IL-1α treatment was continued concurrently. As shown in Figure 3A,B, dexamethasone reduced the mRNA expression and protein levels of genes associated with inflammatory responses such as *IL-6* and *COX2*, synthesis of metalloproteases such as *MMP2* and *MMP13* and genes associated with hypertrophy such as *bFGF*, *COL3A1* and *TNFRSF11b*.

### 2.3. Analysis of the Effect of Dexamethasone in Ligamentum Flavum Cell Fibrosis

**Figure 4:**
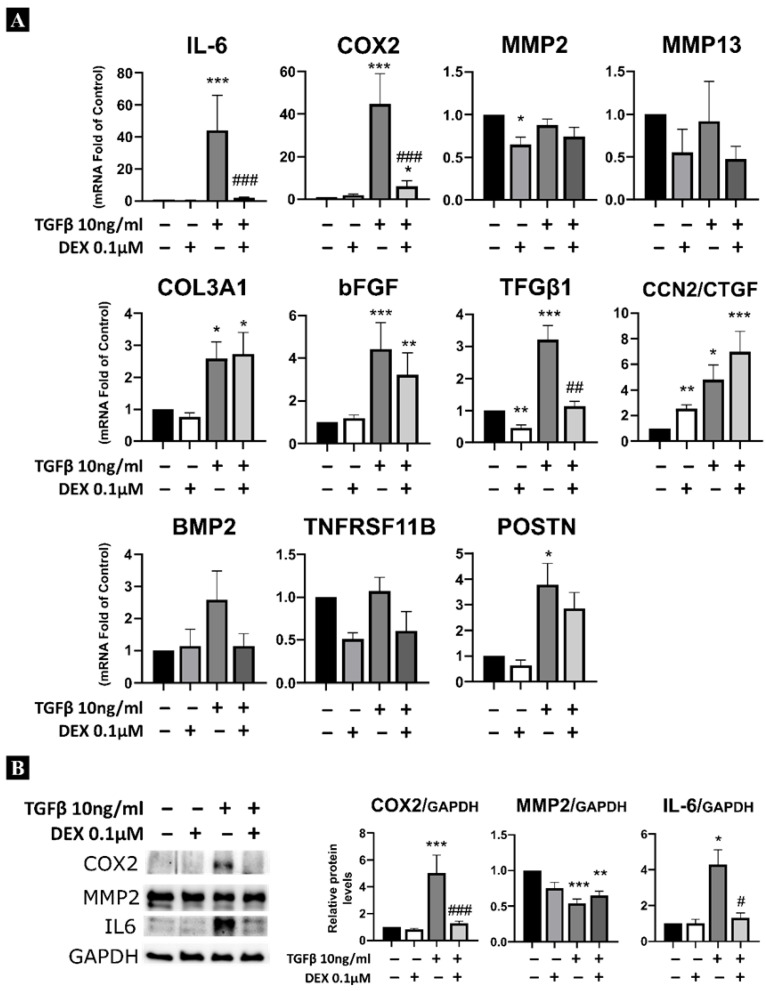
Analysis of the inhibitory capacity of dexamethasone in ligamentum flavum cells treated with TGFβ1. (**A**) Analysis of mRNA levels of cells treated with TGFβ1 (10 ng/mL) and/or dexamethasone (0.1 µM). (**B**) Analysis of protein expression levels of cells treated with TGFβ1 and/or dexamethasone. Statistical significance is represented as follows: * *p* < 0.05, ** *p* < 0.01, *** *p* < 0.001 compared with control expression levels; # *p* < 0.05, ## *p* < 0.01, ### *p* < 0.001 compared with TGFβ1 treatment.

As shown in Figure 4, TGFβ1 was able to induce gene expression of inflammatory markers such as *IL-6* and *COX2*; fibrotic markers such as *COL3A1* and *bFGF*; ossification markers such as *BMP2* and *POSTN*; and fibrotic factors such as *TGFβ1* and *CTGF*/*CCN2*. Notably, TGFβ1 treatment did not induce metalloprotease (*MMP2* and *MMP9*) and *TNFRSF11B* expression.

Dexamethasone addition reduced the TGFβ1-induced expression of genes associated with inflammatory response such as *IL-6* and *COX2*, in addition to *TGFβ1* and *BMP2*. However, no significant reduction was observed in the mRNA expression of fibrosis-related genes such as *COL3A1*, *bFGF*, and *POSTN*. Moreover, *CTGF*/*CCN2* mRNA expression levels increased during dexamethasone treatment alone, further enhancing the induction when combined with TGFβ1 treatment.

## 3. Discussion

Glucocorticoids are extensively utilised in the treatment of various inflammatory disorders, including asthma, cystic fibrosis, and rheumatoid arthritis, owing to their strong anti-inflammatory effects [17]. In cases of spinal canal stenosis, corticosteroids, administered either epidurally or systemically, are prescribed for patients who do not require surgical intervention. Nonetheless, recent research has suggested that these therapies may be limited in their effectiveness at relieving pain associated with canal stenosis, particularly over extended periods [18,19,20]. The challenge is further compounded by the limited understanding of the molecular mechanisms through which glucocorticoids exert their effects in LFH. To the best of our knowledge, the present study is the first to evaluate corticosteroid action, specifically dexamethasone, on the inflammation and fibrotic process of the ligamentum flavum cells.

In LF cells, dexamethasone robustly suppresses the acute inflammatory response triggered through IL-1R and TLR4 activation, mirroring its effects in other tissues [21,22]. Beyond its anti-inflammatory activity, dexamethasone treatment also supresses the expression of metalloproteinases (*MMP2* and *MMP13*) as well as fibrotic markers such as *COL3A1* and *bFGF*, which are involved in LF remodelling [23,24]. Moreover, it reduces the expression of ossification-related genes, including *RUNX2*, *BMP2*, and *TNFRSF11B*, which are typically elevated during LF hypertrophy [25,26]. Notably, dexamethasone’s effects are not confined to the early stages of inflammation. It continues to suppress the expression of these pro-inflammatory, fibrotic, and ossification markers even during sustained inflammatory conditions. These results indicate that dexamethasone may not only hinder the progression of ligamentum flavum hypertrophy by suppressing inflammation, but may also hold the potential to counteract established inflammatory processes within the tissue.

Elevated levels of *TGFβ1* have been observed in hypertrophic LF tissue, where this cytokine is thought to play a pivotal role in driving pathological remodelling [27]. In vitro studies have shown that stimulation of LF cells with exogenous TGFβ1 leads to a significant upregulation of both inflammatory markers, such as *IL-6* and *COX2*, and fibrotic markers, including *COL3A1*, *bFGF*, and *POSTN* [28,29]. These findings underscore the dual function of TGFβ1 as both a pro-inflammatory and pro-fibrotic factor in the remodelling of ligamentum flavum tissue. Interestingly, TGFβ1 also appears to induce a self-sustaining loop through autocrine signalling. The exogenous administration of TGFβ1 has been shown to stimulate its own gene expression in LF cells, suggesting a positive feedback mechanism that may contribute to the chronic activation of fibrogenic pathways. This hypothesis is supported by immunohistochemical analyses of hypertrophic LF tissue, which frequently demonstrate cytoplasmic accumulation of TGFβ1 within fibroblasts, indicative of persistent and dysregulated production of this cytokine [30].

When evaluating the modulatory effects of dexamethasone on TGFβ1-induced responses, a differential pattern emerges. Dexamethasone significantly attenuates the expression of inflammatory genes induced by TGFβ1, confirming its robust anti-inflammatory action even in a profibrotic environment. However, in contrast to its suppression of inflammation, dexamethasone fails to significantly reduce the expression of key fibrosis-associated genes, including *COL3A1*, *bFGF*, and *POSTN*. This is in line with previous studies on fibroblasts from other tissues, where glucocorticoids likewise failed to effectively suppress the expression of fibrotic markers such as *ACTA2*, *FN1*, and *COL1A1* [31]. Finally, although dexamethasone effectively counteracts inflammation-mediated fibrosis, it may have limited efficacy in counteracting fibrogenesis initiated by non-inflammatory stimuli such as TGFβ1.

Another significant observation is the increase in *CTGF*/*CCN2* mRNA expression following treatment with dexamethasone alone, which was further amplified when combined with TGFβ1 treatment. *CTGF*/*CCN2* is a well-established mediator of fibrosis and tissue remodelling, often acting downstream of TGFβ signalling [32]. In line with these results, the treatment with dexamethasone was observed to upregulate pro-fibrotic markers in other tissues [33,34,35]. These results raise the possibility that, under certain circumstances, dexamethasone may promote fibrotic remodelling despite its well-known anti-inflammatory properties. Therefore, although dexamethasone remains an effective anti-inflammatory agent, its potential to contribute to fibrosis in specific situations highlights the need for caution when considering long-term or repeated use in the treatment of LFH. This observation may also have clinical implications, as epidural corticosteroids are widely used to reduce inflammation and alleviate symptoms in degenerative spine disorders [36]. Repeated exposure to glucocorticoids could potentially contribute to fibrotic changes in the ligamentum flavum microenvironment, as we observed in *CTGF*/*CCN2* levels, potentially facilitating the progression of ligamentum flavum hypertrophy. Further experiments and clinical studies are required to clarify the paradoxical fibrogenic effect of dexamethasone in LFH management. In conclusion, dexamethasone exerts potent anti-inflammatory effects in LF cells but its impact on fibrotic remodelling appears to be limited, particularly when fibrosis is driven by non-inflammatory mechanisms.

These findings provide valuable insights that could inform the rational use of glucocorticoids in LFH, highlighting the importance of concurrently targeting inflammatory and fibrotic pathways to enhance therapeutic efficacy. Given that LFH involves both inflammatory and non-inflammatory fibrotic components, a singular reliance on corticosteroids might be insufficient to halt the long-term progression of lumbar spinal stenosis. Therefore, further research into a ‘dual-target’ therapeutic strategy that combines steroids with anti-fibrotic agents is needed. Potential targets could include TGF-β receptor inhibitors, CTGF-neutralising antibodies, or SMAD signalling inhibitors. Potential risks of pathway over-suppression should be carefully considered. Excessive inhibition of TGF-β/SMAD or CTGF signalling may impair normal extracellular matrix turnover and physiological wound healing, potentially leading to delayed or impaired tissue regeneration. Moreover, combined suppression of inflammatory and fibrotic pathways could disrupt normal immune homeostasis. This study has several limitations inherent to its in vitro design. Firstly, although the use of primary LF cells is valuable, it does not fully replicate the complex cellular interactions and microenvironment found in vivo such as interactions with immune cells, vascular components, and mechanical stress. Moreover, fibrosis in vivo develops through prolonged and multifactorial signalling networks, whereas our experimental system relies on simplified cytokine stimulation. Consequently, the effects observed may not completely represent the physiological context of ligamentum flavum hypertrophy. Furthermore, we believe that caution is warranted when translating these findings clinically, as systemic or repeated local corticosteroid administration is associated with adverse effects and inconsistent efficacy in patients. Future research involving in vivo models and clinical specimens is essential to validate these results and to investigate combination therapies that more effectively address both the inflammatory and fibrotic aspects of ligamentum flavum hypertrophy.

## 4. Materials and Methods

### 4.1. Cell Cultures

Ligamentum flavum (LF) tissue samples were obtained from patients undergoing spinal surgeries (Table S1). Written informed consent was obtained from all patients enrolled in the study. Ethical approval was granted by local ethical committee. Each sample was cut into small fragments and digested for 30 min with Pronase (Promega, Madison, WI, USA), followed by an 8 h digestion with Collagenase (Promega, Madison, WI, USA). The resulting cell suspensions were filtered using a 40 μm cell strainer and cultured at 37 °C in a humidified atmosphere containing 5% CO_2_ until reaching 70–90% confluence in DMEM/F-12 (Corning, Corning, NY, USA) supplemented with 10% foetal bovine serum (FBS; Hyclone, Holland), 2 mM L-glutamine, and antibiotics (penicillin 50 units/mL and streptomycin 50 μg/mL). All cells used in the following experiments were below passage number 6. Cell phenotype verification throughout the passages was performed by qPCR, analysing specific LF cell markers, such as *COL3A1*, *NC* and *SCX* (Figure S1).

Then, 200,000 cells/well were seeded in 6-well plates to approximately 75% confluence. The 24 h experiments were performed after a 4 h starvation period in FBS-free conditions. Longer-duration experiments, more than 24 h, were performed in a 5% FBS-supplemented medium. LPS, IL-1α and IL-1β from Sigma-Aldrich (St. Louis, MO, USA), TGFβ1 from Thermo Fisher Scientific (Waltham, MA, USA) and dexamethasone (DEX) from Kern Pharma (Carnaxide, Portugal) were used to treat the cells.

### 4.2. RNA Extraction and Real-Time Reverse Transcription–Polymerase Chain Reaction (RT-qPCR)

Total RNA was isolated from LF cells using NZYol (NZYTech, Lisbon, Portugal) and E.Z.N.A. Total RNA Kit I (Omega Bio-tek, Norcross, GA, USA) according to the manufacturer’s instructions. Reverse transcription was performed using NZY First-Strand cDNA Synthesis Kit (NZYTech, Portugal). Real-time qPCR was performed using RT2 SYBR Green qPCR Mastermix (Qiagen, Hilden, Germany) in an AriaMx Real-time PCR System (Agilent Technologies, Santa Clara, CA, USA) according to the manufacturer’s instructions. The primers used are listed in Table 1. Quantitative results were calculated using the comparative ΔΔCT method and using ARIA MX software (V. 2.1.1, Agilent, Santa Clara, CA, USA).

### 4.3. Western Blot Analysis

Proteins were extracted using lysis buffer (10 mM Tris/HCl, pH 7.5, 5 mM EDTA, 150 mM NaCl, 30 mM sodium pyrophosphate, 50 mM sodium fluoride, 1 mM sodium orthovanadate, 0.5% Triton X-100, 1 mM PMSF, and protease inhibitor cocktail from Thermo Fisher Scientific (Waltham, MA, USA)) and prepared for Western blot as previously described [37]. Primary antibodies against COX2 (1:1000), IL-6 (1:1000) from Cell Signaling Technology (Danvers, MA, USA), MMP2(1:1000, GeneTex, Irvine, CA, USA) and GAPDH (1:2000; Sigma-Aldrich, Saint Louis, MO, USA) were incubated overnight at 4 °C. Later, appropriate secondary antibodies, goat anti-rabbit IgG HRP (1:5000) from GE Healthcare (Chicago, IL, USA), and proteins were detected using Immobilon Western Detection kit (Millipore, Burlington, MA, USA). The images were captured with ChemiDoc MP Imaging System and analysed with Image Lab 6.0.1 Software, both from Bio-Rad Laboratories (Hercules, CA, USA).

### 4.4. Statistical Analysis

Statistical analyses were performed using GraphPad Prism 9.3.1 (Software, https://www.graphpad.com). Data from at least four independent experiments using LF cells from different patients were analysed using one-way analysis of variance (ANOVA), followed by Bonferroni multiple comparison test. A *p*-value less than 0.05 was considered statistically significant. Data are shown at graphics as mean ± SEM.

## Figures and Tables

**Table 1:** The primer sequences used in RT-QPCR.

Gene	Forward	Reverse	Amplicon (bp)
*ACTB*	ACAGAGCCTCGCCTTTGC	GATATCATCATCCATGGTGAGCTGG	70 bp
*bFGF*	AAGAGCGACCCTCACATCAA	ACGGTTAGCACACACTCCTT	81 bp
*BMP2*	TGTATCGCAGGCACTCAGGTCA	CCACTCGTTTCTGGTAGTTCTTC	133 bp
*CCN2/CTGF*	GGAGTGGGTGTGTGACGAG	GTCTTCCAGTCGGTAAGCCG	73 bp
*COL3A1*	GAAAGAGGATCTGAGGGCTCC	AAACCGCCAGCTTTTTCACC	137 bp
*ELF3*	Qiagen (PPH09786A)		
*GAPDH*	Qiagen (PPH00150F)		
*MMP13*	ACTGAGAGGCTCCGAGAAATG	GAACCCCGCATCTTGGCTT	103 bp
*MMP2*	AGCGAGTGGATGCCGCCTTTAA	CATTCCAGGCATCTGCGATGAG	138 bp
*POSTN*	GACCAAGGAAAACTCACTAC	CTGTTTAACTGGTATGGCAC	84 bp
*PTGS2/COX2*	Qiagen (PPH01136F)		
*RUNX2*	TCGCCTCACAAACAACCACA	GCTTGCAGCCTTAAATGACTCT	144 bp
*TGFβ1*	GGCCAGATCCTGTCCAAGC	GTGGGTTTCCACCATTAGCAC	201 pb
*TNFRSF11b*	CCAAGCCCCTGAGGTTTCC	GATGTCCAGAAACACGAGCG	71 bp

## Data Availability

The original contributions presented in this study are included in the article/Supplementary Materials. Further inquiries can be directed to the corresponding authors.

## Supplementary Materials

The following supporting information can be downloaded at https://www.mdpi.com/article/10.3390/ijms27073047/s1.

## Abbreviations

The following abbreviations are used in this manuscript:

ACTA2	Actin Alpha 2, Smooth Muscle
bFGF	Basic Fibroblast Growth Factor
BMP2	Bone Morphogenetic Protein 2
CCN2/CTGF	Cellular Communication Network Factor 2/Connective Tissue Growth Factor
PTGS2/COX2	Prostaglandin-endoperoxide synthase 2 (Cyclooxygenase-2)
COL3A1	Collagen Type III Alpha 1 Chain
ELF3	E74 Like ETS Transcription Factor 3
FN1	Fibronectin 1
GAPDH	Glyceraldehyde-3-Phosphate Dehydrogenase
IL-1α	Interleukin-1 Alpha
IL-1β	Interleukin-1 Beta
IL-6	Interleukin-6
LF	Ligamentum Flavum
LFH	Ligamentum Flavum Hypertrophy
LFC	Ligamentum Flavum Cells
LPS	Lipopolysaccharide
LSS	Lumbar Spinal Stenosis
MMP2	Matrix Metalloproteinase-2
MMP9	Matrix Metalloproteinase-9
MMP13	Matrix Metalloproteinase-13
POSTN	Periostin
qPCR/RT-qPCR	Quantitative Polymerase Chain Reaction/Reverse Transcription Quantitative PCR
RUNX2	Runt-Related Transcription Factor 2
TGFβ/TGFβ1	Transforming Growth Factor Beta/Transforming Growth Factor Beta 1
TLR4	Toll-Like Receptor 4
TNFRSF11B	Tumour Necrosis Factor Receptor Superfamily Member 11B

## References

[1] Sairyo K., Biyani A., Goel V., Leaman D., Booth R., Thomas J., Gehling D., Vishnubhotla L., Long R., Ebraheim N. (2005). Pathomechanism of Ligamentum Flavum Hypertrophy: A Multidisciplinary Investigation Based on Clinical, Biomechanical, Histologic, and Biologic Assessments. Spine.

[2] Siebert E., Prüss H., Klingebiel R., Failli V., Einhäupl K.M., Schwab J.M. (2009). Lumbar spinal stenosis: Syndrome, diagnostics and treatment. Nat. Rev. Neurol..

[3] Viejo-Fuertes D., Liguoro D., Rivel J., Midy D., Guerin J. (1998). Morphologic and histologic study of the ligamentum flavum in the thoraco-lumbar region. Surg. Radiol. Anat..

[4] Sugimoto K., Nakamura T., Tokunaga T., Uehara Y., Okada T., Taniwaki T., Fujimoto T., Mizuta H. (2018). Matrix metalloproteinase promotes elastic fiber degradation in ligamentum flavum degeneration. PLoS ONE.

[5] Zhai J., Guo S., Li J., Chen B., Zhao Y. (2022). Progression of Spinal Ligament Ossification in Patients with Thoracic Myelopathy. Orthop. Surg..

[6] Zhang B., Chen G., Yang X., Fan T., Chen X., Chen Z. (2021). Dysregulation of MicroRNAs in Hypertrophy and Ossification of Ligamentum Flavum: New Advances, Challenges, and Potential Directions. Front. Genet..

[7] Löhr M., Hampl J.A., Lee J.Y., Ernestus R.I., Deckert M., Stenzel W. (2011). Hypertrophy of the lumbar ligamentum flavum is associated with inflammation-related TGF-β expression. Acta Neurochir..

[8] Sairyo K., Biyani A., Goel V.K., Leaman D.W., Booth R., Thomas J., Ebraheim N.A., Cowgill I.A., Mohan S.E. (2007). Lumbar Ligamentum Flavum Hypertrophy Is Due to Accumulation of Inflammation-Related Scar Tissue. Spine.

[9] Nakatani T., Marui T., Hitora T., Doita M., Nishida K., Kurosaka M. (2002). Mechanical stretching force promotes collagen synthesis by cultured cells from human ligamentum flavum via transforming growth factor-β1. J. Orthop. Res..

[10] Chao Y.H., Tsuang Y.H., Sun J.S., Sun M.G., Chen M.H. (2012). Centrifugal force induces human ligamentum flavum fibroblasts inflammation through activation of JNK and p38 pathways. Connect. Tissue Res..

[11] Moon H.J., Park Y.K., Ryu Y., Kim J.H., Kwon T.H., Chung H.S., Kim J.H. (2012). The angiogenic capacity from ligamentum flavum subsequent to inflammation: A critical component of the pathomechanism of hypertrophy. Spine.

[12] Sun C., Zhang H., Wang X., Liu X. (2020). Ligamentum flavum fibrosis and hypertrophy: Molecular pathways, cellular mechanisms, and future directions. FASEB J..

[13] Lurie J., Tomkins-Lane C. (2016). Management of lumbar spinal stenosis. BMJ.

[14] Lee B.H., Moon S.H., Suk K.S., Kim H.S., Yang J.H., Lee H.M. (2020). Lumbar Spinal Stenosis: Pathophysiology and Treatment Principle: A Narrative Review. Asian Spine J..

[15] Ramamoorthy S., Cidlowski J.A. (2016). Corticosteroids. Mechanisms of Action in Health and Disease. Rheum. Dis. Clin. N. Am..

[16] Manchikanti L., Cash K.A., McManus C.D., Pampati V., Abdi S. (2008). Preliminary results of a randomized, equivalence trial of fluoroscopic caudal epidural injections in managing chronic low back pain: Part 4—Spinal stenosis. Pain Physician.

[17] Barnes P.J. (2006). How corticosteroids control inflammation: Quintiles Prize Lecture 2005. Br. J. Pharmacol..

[18] Chou R., Pinto R.Z., Fu R., Lowe R.A., Henschke N., Dana T. (2016). Systemic corticosteroids for radicular and non-radicular low back pain. Cochrane Database Syst. Rev..

[19] Rodrigues L.C.L., Natour J. (2014). A double-blind, randomized controlled, prospective trial assessing the effectiveness of oral corticoids in the treatment of symptomatic lumbar canal stenosis. J. Negat. Results Biomed..

[20] Akbari Aghdam H., Andalib A., Asadiyan Ardakani H., Telloo M., Sheikhbahaei E. (2020). A short-term oral corticosteroid for refractory lumbar spinal stenosis: A double-blinded randomized placebo-controlled clinical trial. Int. J. Rehabil. Res..

[21] Patil R.H., Naveen Kumar M., Kiran Kumar K.M., Nagesh R., Kavya K., Babu R.L., Ramesh G.T., Sharma S.C. (2018). Dexamethasone inhibits inflammatory response via down regulation of AP-1 transcription factor in human lung epithelial cells. Gene.

[22] Chen Y., Zhang C., Xiao C.X., Li X.D., Hu Z.L., He S.D., Xiao X.J., Xu F. (2021). Dexamethasone can attenuate the pulmonary inflammatory response via regulation of the lncH19/miR-324-3p cascade. J. Inflamm..

[23] Fei C., Chen Y., Tan R., Yang X., Wu G., Li C., Shi J., Le S., Yang W., Xu J. (2025). Single-cell multi-omics analysis identifies SPP1+ macrophages as key drivers of ferroptosis-mediated fibrosis in ligamentum flavum hypertrophy. Biomark. Res..

[24] Chuang H.C., Tsai K.L., Tsai K.J., Tu T.Y., Shyong Y.J., Jou I.M., Hsu C.-C., Shih S.-S., Liu Y.-F., Lin C.-L. (2020). Oxidative stress mediates age-related hypertrophy of ligamentum flavum by inducing inflammation, fibrosis, and apoptosis through activating Akt and MAPK pathways. Aging.

[25] Uchida K., Yayama T., Cai H.X., Nakajima H., Sugita D., Guerrero A.R., Kobayashi S., Yoshida A., Chen K.-B., Baba H. (2011). Ossification process involving the human thoracic ligamentum flavum: Role of transcription factors. Arthritis Res. Ther..

[26] Hou X.F., Fan D.W., Sun C.G., Chen Z.Q. (2014). Recombinant human bone morphogenetic protein-2-induced ossification of the ligamentum flavum in rats and the associated global modification of histone H3. J. Neurosurg. Spine.

[27] Wang S., Qu Y., Fang X., Ding Q., Zhao H., Yu X., Xu T., Lu R., Jing S., Liu C. (2023). Decorin: A potential therapeutic candidate for ligamentum flavum hypertrophy by antagonizing TGF-β1. Exp. Mol. Med..

[28] Yang K., Chen Y., Xiang X., Lin Y., Fei C., Chen Z., Lai Z., Yu Y., Tan R., Dong J. (2022). EGF Contributes to Hypertrophy of Human Ligamentum Flavum via the TGF-β1/Smad3 Signaling Pathway. Int. J. Med. Sci..

[29] Ye S., Kwon W.K., Bae T., Kim S., Lee J.B., Cho T.H., Park J., Kim K., Hur J.K., Hur J.W. (2019). CCN5 Reduces Ligamentum Flavum Hypertrophy by Modulating the TGF-β Pathway. J. Orthop. Res..

[30] Park J.B., Chang H., Lee J.K. (2001). Quantitative Analysis of Transforming Growth Factor-Beta 1 in Ligamentum Flavum of Lumbar Spinal Stenosis and Disc Herniation. Spine.

[31] Nakamura R., Mukudai S., Bing R., Garabedian M.J., Branski R.C. (2020). Complex fibroblast response to glucocorticoids may underlie variability of clinical efficacy in the vocal folds. Sci. Rep..

[32] Sun C., Zhang H., Liu X. (2021). Emerging role of CCN family proteins in fibrosis. J. Cell. Physiol..

[33] Sarsenbayeva A., Pereira M.J., Nandi Jui B., Ahmed F., Dipta P., Fanni G., Almby K., Kristófi R., Hetty S., Eriksson J.W. (2022). Excess glucocorticoid exposure contributes to adipose tissue fibrosis which involves macrophage interaction with adipose precursor cells. Biochem. Pharmacol..

[34] Okada H., Kikuta T., Inoue T., Kanno Y., Ban S., Sugaya T., Takigawa M., Suzuki H. (2006). Dexamethasone induces connective tissue growth factor expression in renal tubular epithelial cells in a mouse strain-specific manner. Am. J. Pathol..

[35] Gan Q., Pan H., Zhang W., Yuan Y., Qian J., Liu C. (2022). Fabrication and evaluation of a BMP-2/dexamethasone co-loaded gelatin sponge scaffold for rapid bone regeneration. Regen. Biomater..

[36] Chou R., Hashimoto R., Friedly J., Fu R., Bougatsos C., Dana T., Sullivan S.D., Jarvik J. (2015). Epidural corticosteroid injections for radiculopathy and spinal stenosis. Ann. Intern. Med..

[37] Conde J., Scotece M., López V., Gómez R., Lago F., Pino J., Gómez-Reino J.J., Gualillo O. (2012). Adiponectin and Leptin Induce VCAM-1 Expression in Human and Murine Chondrocytes. PLoS ONE.

