# Supplementary material for: Novel Molecular Insights into the Anti-Inflammatory and Antifibrotic Effects of Dexamethasone on Human Ligamentum Flavum-Derived Cells
Source: Int J Mol Sci. 2026 Mar 27;27(7):3047. doi: 10.3390/ijms27073047 (PMC13073170; doi:10.3390/ijms27073047)
Supplement: Supplementary file 1 [file ijms-27-03047-s001.zip › ijms-4181017-supplementary.pdf]

Supplementary Material

Table S1. Patient demographics and clinical characteristics.

Summary of demographic and clinical data of patients from whom ligamentum flavum (LF) tissue samples were obtained for primary cell isolation. Data include age distribution, sex, and mean ligamentum flavum thickness. Values are presented as mean ± standard deviation (SD), range, or number of patients with percentage where appropriate.

**Patient's number**		39
**Age**		
	mean	56.2 years
	SD	± 21.8 years
	min	13 years
	max	84 years
	<30 years	6 (15.38%)
	30-60 years	12 (30.77%)
	>60 years	21 (53.85%)
**Gender**		
	Male	18 (46.15%)
	Female	21 /53.85%)
**LF thickness**		4,74+/-1.13mm

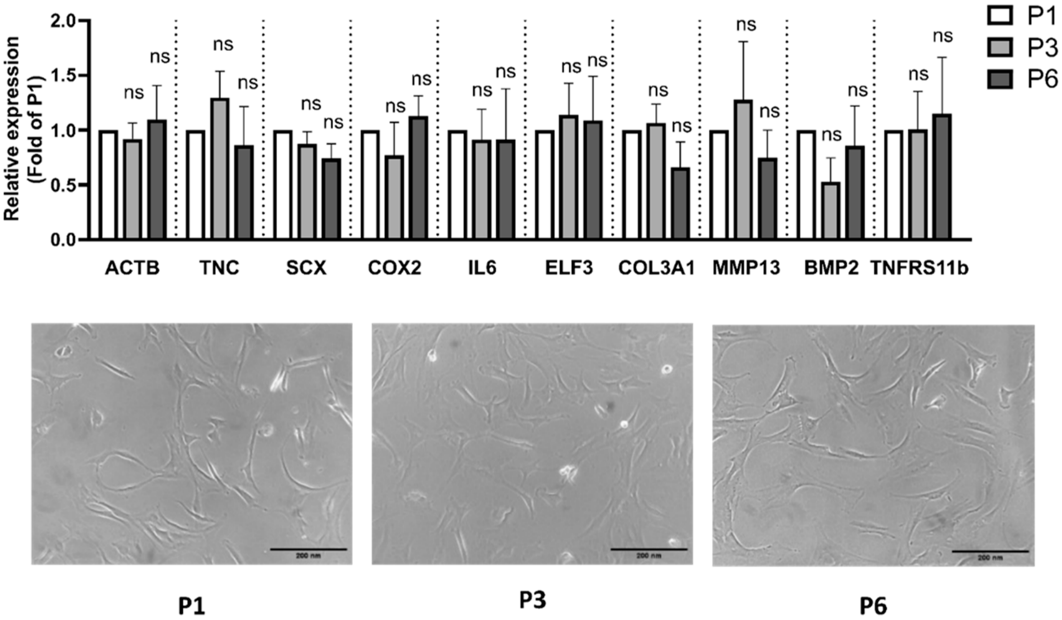

Figure S1. Effect of cell passage on the phenotype of yellow ligament-derived cells. A) Relative mRNA expression levels of different genes. The expression levels of cells derived from different patients at passages 1, 3, and 6 were measured by qPCR and normalized to the expression at passage 1, which is the initial culture obtained from the patients. B) Microscopic images of yellow ligament-derived cells at passages 1, 3, and 6. Images captured with a microscope show yellow ligament-derived cells at passages 1, 3, and 6, with a scale bar equivalent to 200nm.

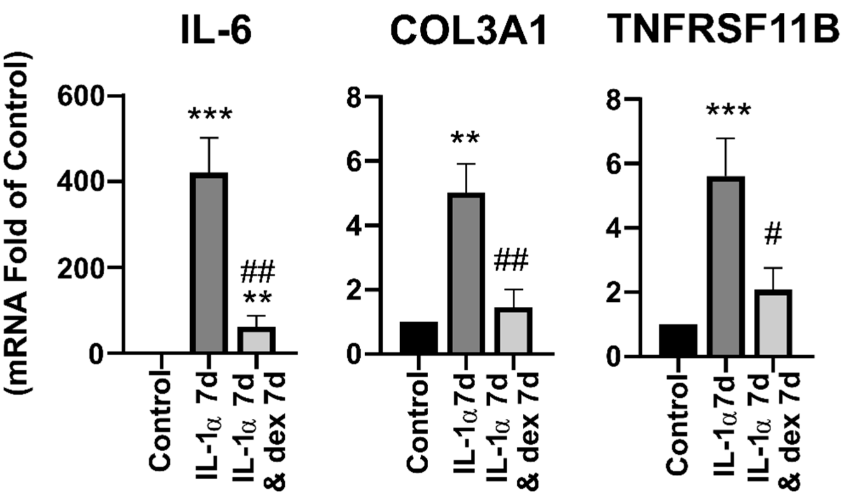

Figure S2. Analysis of the effect of dexamethasone to prevent inflammation in ligamentum flavum-derived cells during prolonged stimulation. Cells were treated with IL-1α for 7 days in the presence or absence of dexamethasone. mRNA (A) and protein (B) expression were analysed. Statistical significance is indicated by * versus control and # versus IL-1α treatment.
